# Differential clinicopathological and molecular features within late-onset colorectal cancer according to tumor location

**DOI:** 10.18632/oncotarget.24502

**Published:** 2018-02-15

**Authors:** Lorena Brandariz, María Arriba, Juan Luis García, Juana María Cano, Daniel Rueda, Eduardo Rubio, Yolanda Rodríguez, Jessica Pérez, Alfredo Vivas, Carmen Sánchez, Sandra Tapial, Laura Pena, Mariano García-Arranz, Damián García-Olmo, Miguel Urioste, Rogelio González-Sarmiento, José Perea

**Affiliations:** ^1^ Surgery Department, Fundación Jiménez Díaz University Hospital, Madrid, Spain; ^2^ Biochemistry Department, Gregorio Marañón University Hospital, Madrid, Spain; ^3^ Molecular Medicine Unit, Department of Medicine, Biomedical Research Institute of Salamanca (IBSAL), Salamanca, Spain; ^4^ Institute of Molecular and Cellular Biology of Cancer (IBMCC), University of Salamanca-SACYL-CSIC, Salamanca, Spain; ^5^ Oncology Department, Ciudad Real General Hospital, Ciudad Real, Spain; ^6^ Molecular Biology Laboratory, “12 de Octubre” Universitary Hospital, Madrid, Spain; ^7^ Digestive Cancer Research Group, “12 de Octubre” Research Institute, Madrid, Spain; ^8^ Surgery Department, “12 de Octubre” Universitary Hospital, Madrid, Spain; ^9^ Pathology Department, “12 de Octubre” Universitary Hospital, Madrid, Spain; ^10^ Familial Cancer Clinical Unit, Spanish National Cancer Centre (CNIO), Madrid, Spain; ^11^ Health Research Institute, Fundación Jiménez Díaz University Hospital, Madrid, Spain; ^12^ Centre for Biomedical Network Research on Rare Diseases (CIBERER), Institute of Health Carlos III, Madrid, Spain

**Keywords:** late-onset colorectal cancer, microsatellite instability, chromosomal instability, CpG island methylator phenotype, colon location

## Abstract

**Background:**

Since there is a predilection of some clinical and molecular features for a given tumor location, we assessed whether this can be confirmed in late-onset colorectal cancer (LOCRC).

**Results:**

Right colon cancers showed features associated with sporadic Microsatellite Instability: predominance of female cases and *BRAF* mutations, and an important mucinous component. Left colon cancers developed a higher number of polyps and multiple primary CRCs, showed the strongest familial component, and had better prognosis. Rectal cancers showed a predominantly sporadic phenotype, with worse prognosis and a CpG Island Methylator Phenotype (CIMP)-High. No copy number alterations (CNAs) greater than or equal to 50% were observed in this LOCRC group, and the most recurrent alterations were losses at 5q13 and 14q11, and gains at 7q11, 7q21-q22, 19p13-p12, 19q13 and 20p11-q11. *KRAS* and *PIK3CA* were the only mutated genes showing differences according to the tumor location, mainly for right colon cancers.

**Materials and Methods:**

We analyzed clinical and molecular characteristics of LOCRC at different tumor locations in order to determine if there are differential phenotypes related with the location in the colon.

**Conclusions:**

Categorizing LOCRC according to tumor location appears to be an adequate first step to resolving the heterogeneity of this subset of CRC.

## INTRODUCTION

Colorectal cancer (CRC) is the second leading cause of cancer-related deaths in western countries [1]. In its pathogenesis, both environmental and genetic factors play an important role. Hereditary CRC syndromes, such as Familial Adenomatous Polyposis (FAP) and Lynch syndrome (LS), are a minority of cases within CRC, and sporadic CRC is the key form. Sporadic CRC mainly arises through the Chromosomal Instability (CIN) pathway, but as the age-of-onset increases the two-other main carcinogenetic pathways, Microsatellite Instability (MSI) and CpG Islands Methylator Phenotype (CIMP), plays increasingly important roles in its development [2]. Moreover, these three main carcinogenetic pathways (CIN, MSI and CIMP) vary according to tumor location, and several studies demonstrate that right colon, left colon and rectal cancers show genetic and molecular differences with divergent underlying carcinogenetic mechanisms and risk factors [3, 4].

Recent studies have also established clinical, genetic and biological differences according to the age-of-onset of CRC, suggesting that it should be a major criterion for subclassifying CRC [5, 6]. Besides, we were able to differentiate two entities within CRC, as early-onset CRC (EOCRC) and late-onset CRC (LOCRC), with variable cut-points in the literature (45 or 50 years old for EOCRC; 70 or 80 years old for LOCRC), being ours 45 and 70, respectively [6]. LOCRC shows an important increase of epigenetic alterations, together with a higher susceptibility for carcinogenesis and a higher global mortality, due to comorbidities and less use of adjuvant treatments in this age group [7]. Nevertheless, it is a group that represents a high proportion within CRC and, as expected, it shares many features with sporadic cases.

To date, there are few studies on CRC that focused specifically on LOCRC and its characteristics, pointing out the need to define clinicopathologic features and prognostic factor that should help to guide treatment decision-making of this subset of CRC. Moreover, apart from those distinct factors according to prognosis, some publications underline the fact that the late-of-onset criteria should not be used when discarding these patients for surgery, improving prognosis for LOCRC patients in most cases [8].

Our group and others have previously defined particular features linked to tumor location in EOCRC [3, 9, 10].

In the present study, we want to apply these same criteria to LOCRC, to examine if tumor location also serves to categorize this subtype better. If so, this will provide a rationale for investigating possible molecular basis for carcinogenesis specific for any of the locations, and thus help to advance the clinical management of this subset of CRC.

## RESULTS

### Global group features

#### Clinicopathological and familial features

Clinical and molecular features of global LOCRC have been published before [6] and are shown in Table 1. According to the tumor location, right colon and rectum lead (39 and 38%, respectively). The remarkable features are the low rate of poor differentiated tumors (4%), but on the other hand, the considerable rate of mucinous component (20%). The higher stage at diagnosis was B (49%), with an outstanding rate of associated polyps through follow-up (64%), mainly adenomatous (61%). Familiar component was low, being most cases sporadic (80%).

**Table 1:** Morphological and clinical description of CRC in LOCRC according to tumor location

	Late-onset CRC *n* (%)	Right Colon *n* (%)	Left Colon *n* (%)	Rectum *n* (%)	*p* (χ^2^)
**Patients**	97 (100)	38 (39.2)	22 (22.7)	37 (38.1)	
**Mean Age at Onset**	77.92	79.08	74.86	78.54	
**(Years, SD)**	(5.73)	(6.01)	(4.96)	(5.36)	**0.015**
**Gender**					
**Male**	50 (51.5)	13 (34.2)	14 (63.6)	23 (62.2)	**0.023**
**Female**	47 (48.5)	25 (65.8)	8 (36.4)	14 (37.8)	
**Grade of differentiation:**					
**Well**	21/90 (23.3)	10/35 (28.6)	5/22 (22.7)	6/33 (18.2)	NS
**Medium**	65/90 (72.2)	22/35 (62.9)	17/22 (77.3)	26/33 (78.8)	
**Poor**	4/90 (4.4)	3/35 (8.6)	0/22 (0)	1/33 (3)	
**Mucosecretion**	18/91 (19.8)	12/35 (34.3)	2/22 (9.1)	4/34 (11.8)	**0.022**
**Signet-Ring Cells**	2/91 (2.2)	0/35 (0)	0/22 (0)	2/34 (5.9)	NS
**Stage (UICC):**					
**I**	5 (5.3)	2 (5.3)	1 (4.5)	2 (5.6)	
**II**	46 (48.9)	22 (57.9)	8 (36.4)	18 (50)	
**III**	24 (25.5)	8 (21)	10 (45.5)	6 (16.7)	
**IV**	19 (20.2)	6 (15.8)	3 (13.6)	10 (27.8)	NS
**Global Survival (months, SD)**	69,83 (11.23)		96,95 (10.26)	31,67 (3.87)	**0.024**
**Disease-Free**		45,94 (4.50)			
**Survival (months, SD)**			31.68 (34.91)	16.25 (14.48)	NS
	21.68 (22.33)	20.16 (14.49)			
**Associated Polyps**	62/97 (63.9)	24/38 (63.2)	13/22 (59.1)	25/37 (67.6)	NS
**Mean number (SD)**	2.70 (4.67)	1.78 (2.17)	5.40 (7.67)	2.0 (3.52)	NS
**Type:**	38/62 (61.3)	13/24 (54.2)	7/13 (53.8)	18/25 (72)	NS
**Adenomatous**	6/62 (9.7)	3/24 (12.5)	0/13 (0)	3/25 (12)	
**Hyperplastic Mixed**	18/62 (29)	8/24 (33.3)	6/13 (46.2)	4/25 (16)	
**Multiple Primary Neoplasms**	33/97 (34)	15/38 (39.5)	9/22 (40.9)	9/37 (24.3)	NS
**Synchronous and/or Metachronous CRCs**	21/97 (21.6)	9/38 (23.7)	8/22 (36.4)	4/37 (10.8)	NS
**Family history of Cancer**					**0.002**
**Amsterdam II**	1/97 (1)	1/38 (2.7)	0/22 (0)	0/37 (0)	
**LS neoplasms**	12/97 (12.4)	6/38 (15.8)	6/22 (27.3)	0/37 (0)	
**Non-LS neoplasms**	6/97 (6.2)	1/38 (2.7)	2/22 (9.1)	3/37 (8.1)	
**Sporadic**	78/97 (80.4)	28/38 (73.7)	12/22 (55.5)	34/37 (91.9)	

Abbreviations: LOCRC: Late-onset Colorectal Cancer. CRC: Colorectal Cancer. SD: Standard Deviation. LS: Lynch Syndrome.

#### Molecular features

Molecular global group features have been published before [6, 30, 28], and summarized in Table 2. Only 9% of cases appeared MSI, mainly associated with *BRAF* mutations. From 90 cases studied for CIMP, the main group appeared was CIMP-0 (49%), and the CIMP-High subgroup was related with the sporadic MSI cases previously described.

**Table 2:** Molecular features of LOCRC according to tumor location

	Late-onset CRC *n* (%)	Right Colon *n* (%)	Left Colon *n* (%)	Rectum *n* (%)	*p* (χ2)
**MSI**	9/97 (9.3)	7/38 (18.4)	2/22 (9.1)	0/37 (0)	**0.023**
**MMR genes mutations**	1/97 (1)	1/38 (2.6)	0/22 (0)	0/37 (0)	NS
**BRAF mutation**	7/97 (7.2)	5/38 (13.2)	1/22 (4.5)	1/37 (2.7)	NS
**CIMP^1^**					
**CIMP-0**	44/90 (48.9)	17/36 (47.2)	14/21 (66.7)	13/33 (39.4)	NS
**CIMP-Low**	24/90 (26.7)	12/36 (33.3)	4/21 (19)	8/33 (24.2)	
**CIMP-High**	22/90 (24.4)	7/36 (19.4)	3/21 (14.3)	12/33 (36.4)	
**Molecular Classification**					**0.002**
**MSI-CIMP-High**	7/90 (7.8)	5/36 (13.9)	2/21 (9.5)	0/33 (0)	
**MSI-CIMP-Low-0**	2/90 (2.2)	2/36 (5.6)	0/21 (0)	0/33 (0)	
**MSS-CIMP-High**	15/90 (16.7)	2/36 (5.6)	1/21 (4.8)	12/33 (36.4)	
**MSS-CIMP-Low-0**	66/90 (73.3)	27/36 (75)	18/21 (85.7)	21/33 (63.6)	
**GENOMIC INSTABILITY^2^**					
**GII**					
**Gains**	0.147055	0.183649	0.101056	0.133332	NS
**Losses**	0.200192	0.249820	0.114539	0.194276	
**Normal**	0.652745	0.566522	0.784362	0.672383	
**Mean of whole altered chromosomes**	3.36	3	1.39	3.76	NS

^1^ Ninety cases were analysed for the global group.

^2^ Eighty-six cases were analysed for the global group.

Abbreviations: LOCRC: Late-onset Colorectal Cancer. MSI: Microsatellite Instability. MMR: Mismatch Repair genes. MSS: Microsatellite Stability. GII: Genomic Instability Index. SD: Standard Deviation.

A total of 86 samples could be adequately processed for Array Comparative Genomic Hybridisation (a-CGH) [30]. The results appeared previously as well, and the most interesting point that should be underlined was that there was no CNA greater or equal to 50% in this group, being the most recurrent alterations: losses at 5q13 and 14q11, and gains at 7q11, 7q21-q22, 19p13-p12, 19q13 and 20p11-q11.

**Figure 1:**
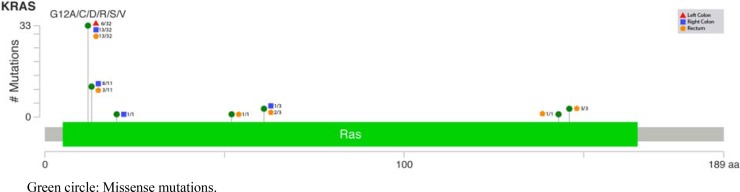
Lollipop plot for KRAS mutations according to the tumor location

**Figure 2:**
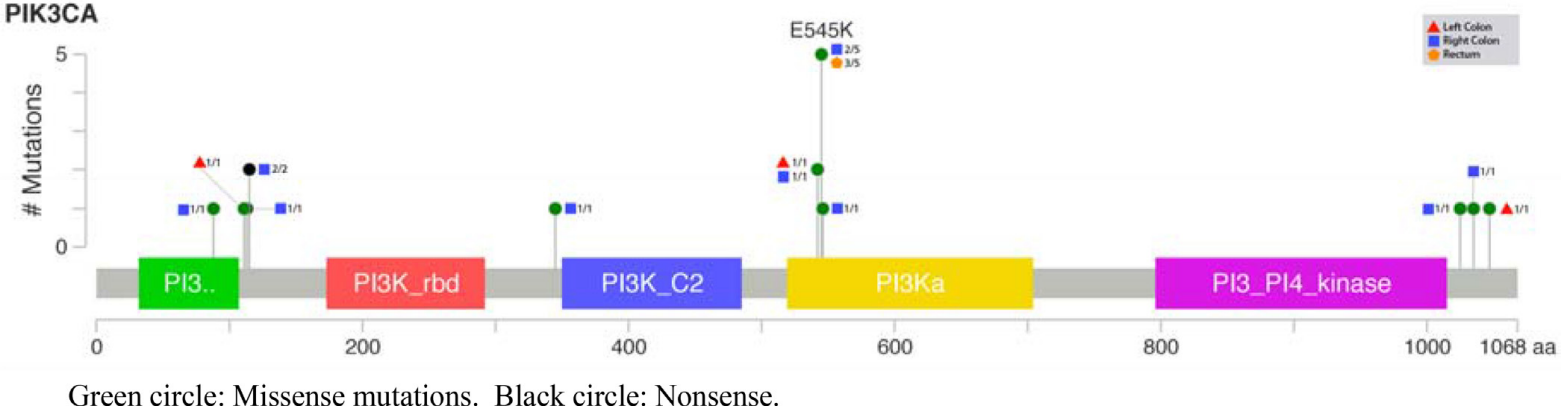
Lollipop plot for PIK3CA mutations according to the tumor location

Finally, both pathogenic mutations and variants of uncertain (or unknown) significance identified by Next Generation Sequencing (NGS) are listed in Supplementary Table 1. Most frequent mutations are those appearing in *KRAS*, *APC* and *p53*, and less frequently in *PIK3CA* (58%, 50%, 45% and 19%, respectively) (Figures 1 and 2; Supplementary Figures 1 and 2); others did not reach the 10%.

### Comparative analysis according to tumor location

#### Clinicopathological and familial features

All these comparative features related with the three main tumor location are summarized in Table 1, being those showing statistical significance described as follows. Comparatively, Right colon cancers were more frequent in females (65.8%), displaying an important proportion of mucinous component (34%). Left colon cancers were younger at age-of-onset (74.86 years-old), with better prognosis, and higher mean number of associated polyps (5.4). Another interesting aspect of this group was the substantial amount of familial cancer component, mainly linked with LS-related neoplasms (27%). Finally, rectal cancers showed sporadic phenotype according to familial cancer history, with worse prognosis: Free Disease Survival (FDS) was minor for rectal locations (Mean: 16.25 months, compared with left colon cancer, 31.68) (*p* = 0.059), as well as the Overall Survival (OS) (mean: 31.67 months, compared with left colon cancers, 31.68 months) (*p* = 0.024). Seventy-five percent of deaths were colorectal cancer-related deaths, mainly due to rectal cancers (52.38%).

#### Molecular features

Everything related with molecular features are described in Table 2. As we have mentioned before, nine cases showed MSI (9.3%), with only one of them due to a MMR-germline mutation and being seven of the others due to *BRAF* mutations and/or Hypermethylation of *MLH1* promoter, most of which were in the Right colon (*p* = 0.023). CIMP-High tumors reached only 24%, with an important amount of them mainly located at the rectum. According to the molecular classification, the most frequent category was MSS-CIMP-Low-0, reaching almost 86% of all Left colon cancers. Interestingly, another important one was MSS-CIMP-High within rectal tumors, with a 36% (*p* = 0.002).

According to the CIN, the location with the largest was right colon, not only talking about Genomic Instability Index (GII), but also in Copy Number Variations (CNV) per cases (data not shown). Meanwhile, left colon cancers show an interesting low Chromosomal Instability (CI), and rectal tumors mannered as an intermediate location, excluding the high mean of whole altered chromosomes. Differences did not show statistical significance maybe due to the intrinsic CIN shown already associated to the aging.

Minimum recurrently lost or gained regions at the different locations are shown in Supplementary Table 2. Moreover, those most frequent altered chromosomal regions for the three cancer locations and the correlative cancer genes codified within are listed in Supplementary Tables 3, 4 and 5. Recurrent gains and losses were more slightly frequent in right-sided LOCRCs, followed by rectal cases. The only recurrent regions that were observed in all three tumor locations were gains of 7q11.22-11.23, and 20q11.1-q11.23. Four regions were common to right- and left-sided LOCRCs: losses at 18q12.1-12.2, 18q21.1 and 18q22.1-23, and gains at 7p22.1. Only two were common to left-sided and rectal LOCRCs: losses at 5p15.33-15.31 and alterations in 1q21.1-21.2. Finally, a high proportion of altered regions were common between right colon and rectal cancers. Individually, the most frequent altered regions for each location were: gains at 7q11.21 and 7q11.23 and losses at 18q22.1-22.3 in right-sided LOCRCs; losses at 10q11.21-11.23 in left-sided LOCRCs; and gains at 7q11.22-11.23 in rectal LOCRCs.

Talking about pathogenic mutations, only *KRAS* and *PIK3CA* showed statistical differences (Supplementary Table 1): *KRAS* appeared scarcely in left colon cancer (29%), while right colon and rectum showed more than 70% and 60%, respectively; and *PIK3CA* showed a decreasing progression from right colon to rectum (33%, 14% and 9%)(Figures 1 and 2).

## DISCUSSION

CRC is a heterogeneous disease with different outcomes and drug responses. Subclassification according to age of onset and tumor location could have applications in terms of diagnosis, prevention and therapy. LOCRC appeared as an important proportion of CRC that is why should be a subset in which focused on. In the general group, without applying location criteria, we found some already-known features, as could be the association with polyp's development during the follow-up, and the sporadic forms predominance. Looking for the main molecular pathways, the low rate of MSI cases, due mainly to sporadic forms, in an intimate relation with CIMP-High have been already published before [11]. The absence of a predominant altered region within the a-CGH analysis, all of which do not reach a 50%, talk about the possible heterogeneity of this group of age. Some of them have been already related with CRC, but in studies without age-of-onset criteria, as loss of 5q13 is [12], or alterations in 7q21, associated with no-responder to neoadjuvant therapy for rectal cancer [13].

As we have mentioned before, we have recently published the differential features for EOCRC according to the tumor location, appearing some interesting subsets within this age-of-onset [3]. In relation with LOCRC, some differences have also emerged. As expected, Right colon cancers fulfilled those features associated with sporadic MSI-CRC [14]: Predominance of female cases and *BRAF* mutations, with an important amount of mucinous component, although, surprisingly, CIMP-High appeared more in rectal cases. While Left colon cancers developed a higher number of polyps and Synchronous and/or Metachronous (SCRCs and/or MCRCs), with a familial cancer component, and better prognosis, maybe related with the high number of polyps’ development, and a more severe follow-up. Rectal cancers show a predominantly sporadic phenotype, with worse prognosis.

Also, it is important to underline the fact of the higher CIN of the Right colon group, in contrast with what it is published, a low CIN due to the MSI component in this location, talking without any age-criterion [15]. This aspect is linked with one interesting point related with the most frequent altered chromosomal segments within LOCRC population, and for every tumor location. Most of them, showed in Supplementary Table 2, are related with some of the identified Common Fragile Sites (FRA) and suggests that these are hotspots of genomic instability leading to inactivation of genes encoded within them, or as that FRA are functional units and that loss of the encoded genes confers selective pressure, leading to cancer development [16]: e.g. the most frequent and shared chromosomal segments by the three colon locations comprises FRA 7B and FRA 20Ad [17, 18]. This may be in relation with the instability component associated with aging exhibited by this subgroup of tumors. Unexpectedly, Left-sided colon cancer appeared as those with the lowest CIN between the three locations. Some other frequent alterations within LOCRC have been already associated with CRC. Gains in 7q11.21, frequent in our right-colon LOCRC, have been associated with LS cases as a whole [19]. Moreover, frequents also in Right colon locations were losses at 18q22.1-22.3, a region whose alteration may be useful as a predictor of benefit from adjuvant fluorouracil therapy [20]. Another, as alteration in 18q21.1, common between right and left colon cancers, has been already connected with a higher risk of CRC [21]. Finally, in one region frequently gained in the three locations, 20q11.1-11.23, is located *SRC*, a proto-oncogene whose variation determine progression in CRC [22].

Most frequent mutations appeared in *KRAS*, *APC* and *p53*, and less frequently in *PIK3CA*, but only the first one overcoming the 50%. A recent study published by Shimada et al. [23], analyzed differences between CRC-without age-of-onset criterion-, according to the tumor location, and within these, studied pathogenic mutations in main genes. They got higher proportions of gene mutations, likening with our results. Nevertheless, although they did not distinct rectal from left colon cancers in the comparative analysis, differences in *PIK3CA* and *KRAS* are correlative with ours, except the high proportion of *KRAS* also seen in rectal location within our population. Other genes, however, did not show differences, compared with the results published by Shimada et al. [23], showing *APC* and *p53* higher rates in right colon cancers, within others. This apparent less importance of pathogenic mutations in most frequent genes, compared with CIN, may be due, between others, to a more important role of this last carcinogenetic pathway in LOCRC, more illustrated by right colon location.

All these findings suggest the importance and in some manner, the need of categorize LOCRC, as we confirmed within EOCRC, according to the tumor location. Not only because phenotypical differences appear from clinical and familial point of views. In the same way that the molecular basis of sporadic MSI cases stays all together in Right-colon category, some specific CNA for each location could be useful as a starting point for future approaches in order to find out specific molecular alterations within the heterogenous group of CRCs, as LOCRC is.

## MATERIALS AND METHODS

### Families, samples and data collection

A total of 100 consecutive individuals with CRC diagnosed at an age of 70 years or older were collected from our institution, starting from January 2002, excluding 3 due to the diagnosis of FAP. They were considered the index case of each family. All patients, or a first degree relative in case of death of the index case, provided written consent. Personal and clinicopathologic information was obtained including age of onset, gender, location of the CRC (right/left colon or rectum), grade of cell differentiation (low, medium or high), mucin production, the presence of “signet ring” cells, modified Astler-Coller stage, the existence of polyps during follow-up, type of polyps (adenomatous, hyperplastic and mixed), the presence of SCRCs or MCRCs, and the presence of primary multiple neoplasms in the index case.

To analyze the antecedents of cancer, families were classified into four groups: a) families fulfilling the Amsterdam II criteria for LS [10]; b) families with mainly aggregation-one in first-degree or two in second-degree family members- of LS-related neoplasms; c) families with mainly aggregation of LS-unrelated neoplasms; d) cases without oncological antecedents; these were considered sporadic cases.

Follow-up was at least 5 years from surgery, defining FD and OS, recurrence and cancer-related death for each case.

### Microsatellite instability and mutational analysis

MSI analysis was performed using the Bethesda panel [24, 25]. The five microsatellite markers were PCR amplified, and fluorescently labeled fragments were evaluated using a 3100-Avant genetic analyzer (Applied Biosystems, Foster City, CA, USA); data were analyzed using GeneMapper software version 3.5 (Applied Biosystems). Tumors were considered as MSI when showing high-frequency MSI (MSI-H) (two or more of the five markers showing instability), while the rest were classified as MSS. MSI cases were screened for germline mutations in the MMR genes *MLH1*, *MSH2* and *MSH6* by denaturing gradient gel electrophoresis using a DCode system (Bio-Rad Laboratories, Hercules, CA, USA), by denaturing high performance liquid chromatography using a Varian ProStar system (Varian Australia Pty Ltd., Melbourne, Australia), or by high-resolution melting analysis using a LightCycler 480 real-time PCR system (Roche, Mannheim, Germany). Primers and denaturing and melting conditions were as previously reported, with slight modifications [26]. When an anomalous band or pattern was observed by denaturing gradient gel electrophoresis, denaturing high-performance liquid chromatography, or high-resolution melting, the PCR product of the fragment was sequenced using the BigDye Terminator v3.1 cycle sequencing kit (Applied Biosystems) and was analyzed using an ABI Prism 3130 genetic analyzer (Applied Biosystems). MSI sporadic cases were identified by determining the methylation status of the *MLH1* gene promoter and/or determination of *BRAF* mutational status. Methods carried out were described in previous publications [6].

### Analysis of CpG island methylation phenotype panel

Methylation status of promoter regions of the CIMP panel genes: *CACNA1G, CDKN2A, CRABP1, IGF2, MLH1, NEUROG1, RUNX3* and *SOCS1* were studied by means of methylation-specific multiplex ligation-dependent probe amplification36 using the SALSA MLPA kit (ME042-B1; MRC-Holland, Amsterdam, The Netherlands) following the manufacturer's recommended procedure. The methylation-specific multiplex ligation-dependent probe amplification products were resolved by capillary electrophoresis using a 3100-Avant genetic analyzer, and peaks were analyzed using GeneMapper software version 3.5. The methylation index at a specific CpG locus was calculated by dividing the normalized area of a given HhaI-digested probe sample and the undigested equivalent. CIMP-High was defined as the presence of ≥ 6/8 methylated promoters, CIMP-Low as 1/8 to 5/8 methylated promoters, and CIMP-0 as the absence (0/8) of methylated promoters.

### Molecular classification

According to the MSI and CIMP status, we classified both groups (early-onset and elderly CRC) into four categories, because differences between MSI-L and MSS are subtle and differences between CIMP-Low and CIMP-0 are also subtle: (MSI/CIMP-High); (MSI/CIMP-Low/0); (MSS/CIMP-High); (MSS/CIMP-Low/0) [27].

### Chromosomal instability: array comparative genomic hybridization (aCGH)

CGH was performed using NimbleGen oligonucleotide microarrays (Roche NimbleGen, Inc., Reykjavik, Iceland) in order to identify CNA, and has been described before. The degree of genomic instability has been already described, as well, in the same work [28], and have been included in the Gene Expression Omnibus (http://www.ncbi.nlm.nih.gov/geo/). Correlative cancer genes codified within those most frequent altered chromosomal regions for the three cancer locations are shown in corresponding tables [29].

### Next generation sequencing (NGS)

#### Ion torrent PGM library preparation

An Ion Torrent adapter-ligated library was generated using the Ion AmpliSeq Library Kit 2.0 and the Ion AmpliSeq Cancer Hotspot Panel version 2 (Thermo Fisher Scientific, Rev. B.0; MAN0006735). Briefly, 2 μL of 5X Ion AmpliSeq™ HiFi mix, 2 μL of 5X Ion AmpliSeq™ Primer Pool and 5 ng of gDNA per reaction were mixed together and amplified following the temperature conditions provided by the manufacturer. Then, primer sequences were partially digested by adding 1 μL of FuPa Reagent and loaded in a thermal cycler under the conditions detailed in the user guide. Finally, each library was marked with a unique adapter provided in Ion Xpress™ barcode adapters 1-96 Kit (Life Technologies) in a reaction mixture containing 2 μL of Switch Solution, 1 μL of diluted barcode and 1 μL of DNA Ligase, also under the temperature conditions provided by the manufacturer.

After AMPure bead (Beckman Coulter, Brea, CA, USA) purification, the concentration of the library (in a 100-fold dilution) was determined using the Ion Library TaqMan quantitation assay kit (Thermo Fisher Scientific) in a 7500 Real-Time PCR System (Thermo Fisher Scientific, Foster City, CA, USA). Each sample was run in a minimum of two replicates.

#### Emulsion PCR

Sample emulsion PCR and enrichment were performed using the Ion PGM™ Template OT2 200 Kit and Ion One Touch™ 2 System (Life Technologies). We followed the manufacturer's instructions except for the concentration of the pooled libraries which in this work was set at 9pM.

#### Sequencing on the Ion torrent PGM platform

All barcoded samples were sequenced using the Ion PGM™ Hi-Q™ Sequencing Kit (Life Technologies) in an Ion Torrent PGM instrument (Life Technologies) with Ion 318™ v2 chips (Life Technologies).

Chip loading procedure was performed according to the user guide for the Ion PGM™ Hi-Q™ Sequencing Kit (Life Technologies). A maximum of 16 samples were loaded on a single chip per sequencing run.

#### Bioinformatics processing and data analysis

Base calling and alignment to the human genome (hg19) were executed with the Torrent Suite Software v.4.0 using the variant caller plugin. Variants were annotated using Ion Reporter and each mutation was verified in the Integrative genome viewer (IGV) from the Broad Institute (http://www.broadinstitute.org/igv/) [30].

### Statistical analyses

Continuous variables were expressed as mean values plus/minus standard desviation (SD), and categorical variables were expressed as number of cases and their percentage. Differences were considered significant when *p* < 0.05. For associations between colon location and other discrete variables, statistical analyses were performed using Pearson's Chi Square (χ^2^). Test for parametric variables, and Fisher's Exact Test for non-parametric variables. When those features were continuous variables, Student's *t* test was used. Survival analyses were also carried out by Kaplan–Meier test. The SPSS v.11.5 for Windows (SPSS, Inc., Chicago, IL, USA) statistical package was used.

For the CGH analysis, statistics were as follows: For identifying significant minimum regions, both univariate and multivariate analysis were carried out. Regarding the univariate analysis, unconditional logistic regression was carried out for each candidate region. For the multivariate analysis, each of the regions was tested separately, including other relevant clinical variables. Location was considered as a factor in all the analysis carried out. This analysis was performed in R Statistics Software [31].

## SUPPLEMENTARY MATERIALS FIGURES AND TABLES











## Abbreviations

RC: colorectal cancer
LOCRC: late-onset colorectal cancer
EOCRC: early-onset colorectal cancer
CIMP: CpG island methylator phenotype
CNA: copy number alterations
FAP: familial adenomatous polyposis
LS: Lynch syndrome
CIN: chromosomal instability
MSI: microsatellite instability
aCGH: array comparative genomic hybridisation
NGS: next generation sequencing
FDS: free disease survival
OS: overall survival
MMR: mismatch repair genes
MSS: microsatellite stability
CNV: copy number variations
CI: chromosomal instability
SCRC: synchronous colorectal cancer
MCRC: metachronous colorectal cancer
IGV: integrative genome viewer
SD: standard deviation
GII: genomic instability index
