# Supplementary material for: Differential clinicopathological and molecular features within late-onset colorectal cancer according to tumor location
Source: Oncotarget. 2018 Feb 15;9(20):15302–11. doi: 10.18632/oncotarget.24502 (PMC5880605; doi:10.18632/oncotarget.24502)
Supplement: Supplementary file 1 [file oncotarget-09-15302-s001.pdf]

Differential clinicopathological and molecular features within late-onset colorectal cancer according to tumor location

SUPPLEMENTARY MATERIALS

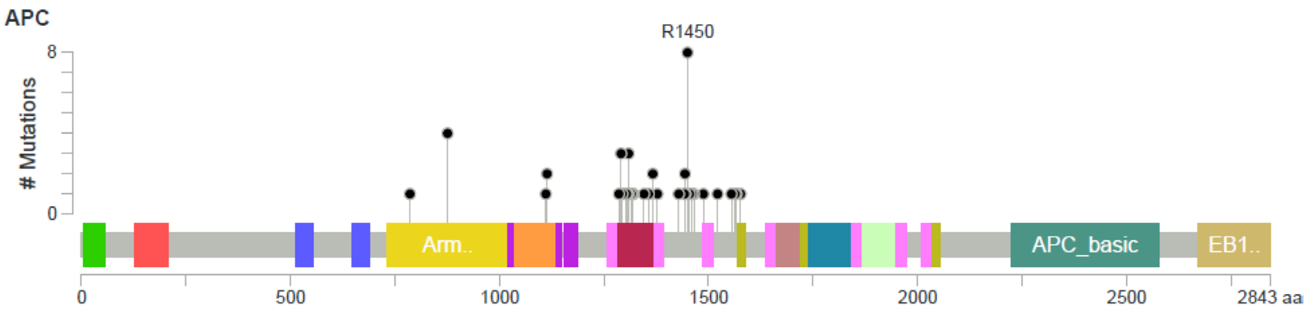

Green circle: Missense mutations. Black circle: Nonsense.

Supplementary Figure 1: Lollipop plot for APC mutations.

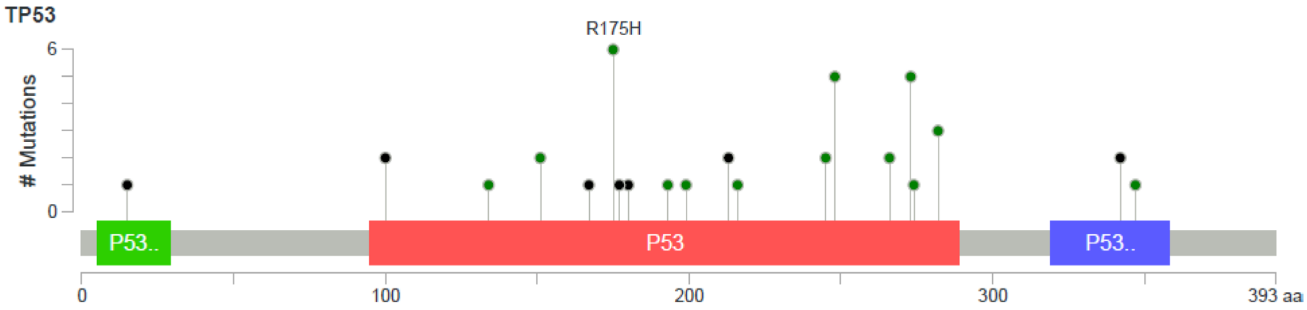

Green circle: Missense mutations. Black circle: Nonsense.

Supplementary Figure 2: Lollipop plot for TP53 mutations.

Supplementary Table 1: Most frequent altered chromosomal regions for each late-onset CRC location. See_Supplementary Table_1

Supplementary Table 2: Pathogenic mutations according to the tumor location

Genes mutations	Late-onset CRC n/Global (%)	Right Colon n/Global (%)	Left Colon n/Global(%)	Rectum n/Global (%)	*p* (χ^2)
**KRAS**	50/86 (58)	22/30 (73)	6/21 (29)	22/35 (63)	**0.005**
**APC**	43/86 (50)	16/30 (53)	7/21 (33)	20/35 (57)	NS
**p53**	39/86 (45)	12/30 (40)	9/21 (43)	18/35 (51)	NS
**PIK3CA**	16/86 (19)	10/30 (33)	3/21 (14)	3/35 (9)	**0.03**
**SMAD4**	8/86 (9)	1/30 (3)	1/21 (5)	6/35 (17)	NS
**FBXW7**	7/86 (8)	1/30 (3)	2/21 (10)	4/35 (11)	NS
**NRAS**	6/86 (7)	2/30 (7)	1/21 (5)	3/35 (9)	NS
**GNAS**	6/86 (7)	4/30 (13)	0	2/35 (6)	NS
**BRAF**	6/86 (7)	3/30 (10)	1/21 (5)	2/35 (6)	NS
**CDKN2A**	3/86 (3)	1/30 (3)	0	2/35 (6)	NS
**PTEN**	2/86 (2)	1/30 (3)	0	1/35 (3)	NS
**Genes with only one location with mutated cases:**					NS
**IDH2**	1/86 (1)	0	0	1/35 (3)	
**CTNNB1**	2/86 (2)	2/30 (7)	0	0	
**SMARCB1**	1/86 (1)	0	0	1/35 (3)	
**KDR**	2/86 (2)	2/30 (7)	0	0	
**CDH1**	1/86 (1)	1/30 (3)	0	0	
**MPL**	1/86 (1)	1/30 (3)	0	0	
**GNAQ**	1/86 (1)	0	0	1/35 (3)	
**GNA11**	1/86 (1)	0	1/21 (5)	0	

Other genes without pathogenic mutations: *AKT1, FGFR3, KIT, RB1, VHL, ERBB4, ATM, FGFR2, HNF1A, NOTCH1, SMO, HRAS, RET, STK11.*
LOCRC: Late-onset Colorectal Cancer. CRC: Colorectal Cancer.

Supplementary Table 3: Most frequent altered chromosomal regions for right colon cancers and the correlative cancer genes codified within. See_Supplementary Table_3

Supplementary Table 4: Most frequent altered chromosomal regions for left colon cancers and the correlative cancer genes codified within. See_Supplementary Table_4

Supplementary Table 5: Most frequent altered chromosomal regions for rectal cancers and the correlative cancer genes codified within. See_Supplementary Table_5
