# Supplementary material for: Differential clinicopathological and molecular features within late-onset colorectal cancer according to tumor location
Source: Oncotarget. 2018 Feb 15;9(20):15302–11. doi: 10.18632/oncotarget.24502 (PMC5880605; doi:10.18632/oncotarget.24502)
Supplement: Supplementary file 2 [file oncotarget-09-15302-s002.docx]

**Supplementary Table 1.** **Most frequent altered chromosomal regions for each late-onset CRC location.**

|  |  |  |  |  |  |  |  |  |  |  |  |
| --- | --- | --- | --- | --- | --- | --- | --- | --- | --- | --- | --- |
| Right colon  Chromosome | Region |  | % | Left colon  Chromosome | Region |  | % | Rectum  Chromosome | Region |  | % |
| chr1 | p32.3 | p31.3 | 49 | chr1 | q21.1 | q21.2 | 32 | chr1 | p36.33 | p36.32 | 38 |
| chr1 | p31.2 | p31.1 | 51 | chr4 | q12 | q13.2 | 32 | chr1 | p36.32 | p36.31 | 41 |
| chr1 | p21.3 | p13.3 | 49 | chr5 | p15.33 | p15.31 | 32 | chr1 | p36.13 | p36.13 | 38 |
| chr1 | p21.3 | p21.3 | 51 | chr7 | p22.1 | p22.1 | 32 | chr1 | p35.3 | p35.1 | 38 |
| chr1 | p21.2 | p21.1 | 51 | chr7 | q11.22 | q11.23 | 32 | chr1 | p35.2 | p35.1 | 41 |
| chr2 | p25.3 | p25.1 | 49 | chr7 | q11.23 | q11.23 | 37 | chr1 | p33 | p33 | 38 |
| chr2 | p25.3 | p25.2 | 51 | chr8 | p22 | p22 | 32 | chr1 | p32.3 | p31.3 | 38 |
| chr2 | q14.3 | q14.3 | 49 | chr10 | q11.21 | q11.23 | 42 | chr1 | p32.3 | p31.1 | 41 |
| chr2 | q22.1 | q22.1 | 49 | chr10 | q26.13 | q26.3 | 32 | chr1 | p31.2 | p31.1 | 38 |
| chr3 | p14.1 | p13 | 49 | chr10 | q26.2 | q26.3 | 37 | chr1 | p21.3 | p13.3 | 38 |
| chr4 | p16.1 | p15.2 | 49 | chr14 | q11.2 | q21.3 | 32 | chr1 | q21.1 | q21.2 | 38 |
| chr4 | q34.3 | q35.2 | 51 | chr14 | q22.1 | q32.2 | 32 | chr2 | p16.1 | p16.1 | 38 |
| chr7 | p22.1 | p22.1 | 51 | chr14 | q32.13 | q32.2 | 37 | chr3 | p26.3 | p26.2 | 38 |
| chr7 | p12.1 | p11.2 | 49 | chr16 | p12.2 | p11.2 | 32 | chr3 | p26.1 | p26.1 | 38 |
| chr7 | p11.2 | p11.2 | 54 | chr16 | p11.2 | p11.2 | 37 | chr3 | p13 | p12.1 | 38 |
| chr7 | q11.21 | q11.21 | 60 | chr16 | q21 | q21 | 32 | chr3 | p11.1 | p11.1 | 38 |
| chr7 | q11.21 | q11.23 | 51 | chr18 | q12.1 | q12.2 | 32 | chr4 | p16.1 | p16.1 | 41 |
| chr7 | q11.21 | q11.22 | 49 | chr18 | q21.1 | q21.1 | 32 | chr4 | p16.1 | p15.31 | 44 |
| chr7 | q11.22 | q11.23 | 57 | chr18 | q22.1 | q23 | 32 | chr4 | p15.33 | p15.1 | 41 |
| chr7 | q11.23 | q11.23 | 60 | chr20 | q11.1 | q11.23 | 32 | chr4 | p15.32 | p14 | 38 |
| chr7 | q21.2 | q21.2 | 51 | chr20 | q12 | q13.33 | 32 | chr5 | p15.33 | p15.31 | 41 |
| chr7 | q21.2 | q22.1 | 54 |  |  |  |  | chr5 | q13.2 | q13.2 | 41 |
| chr7 | q21.2 | q22.3 | 49 |  |  |  |  | chr5 | q31.1 | q31.2 | 38 |
| chr7 | q21.3 | q22.3 | 57 |  |  |  |  | chr7 | p11.2 | p11.2 | 41 |
| chr7 | q22.1 | q22.1 | 60 |  |  |  |  | chr7 | q11.21 | q11.21 | 38 |
| chr7 | q22.1 | q22.3 | 54 |  |  |  |  | chr7 | q11.21 | q11.22 | 41 |
| chr7 | q22.1 | q32.2 | 51 |  |  |  |  | chr7 | q11.22 | q11.23 | 56 |
| chr7 | q32.1 | q32.3 | 49 |  |  |  |  | chr7 | q22.1 | q22.1 | 41 |
| chr7 | q33 | q34 | 49 |  |  |  |  | chr9 | p13.1 | p13.1 | 41 |
| chr8 | q24.12 | q24.21 | 49 |  |  |  |  | chr9 | p13.1 | p11.2 | 38 |
| chr9 | p13.1 | p13.1 | 49 |  |  |  |  | chr9 | q12 | q13 | 38 |
| chr9 | p11.2 | p11.2 | 49 |  |  |  |  | chr13 | q21.33 | q22.1 | 38 |
| chr9 | q12 | q13 | 51 |  |  |  |  | chr13 | q32.1 | q32.3 | 41 |
| chr9 | q13 | q21.11 | 49 |  |  |  |  | chr13 | q32.1 | q33.3 | 38 |
| chr15 | q25.2 | q26.1 | 49 |  |  |  |  | chr13 | q34 | q34 | 38 |
| chr15 | q26.1 | q26.2 | 51 |  |  |  |  | chr16 | p13.12 | p13.11 | 41 |
| chr16 | p13.11 | p13.11 | 49 |  |  |  |  | chr16 | p13.11 | p11.2 | 44 |
| chr16 | p13.11 | p12.3 | 51 |  |  |  |  | chr16 | p12.3 | p11.2 | 38 |
| chr18 | p11.32 | p11.31 | 54 |  |  |  |  | chr18 | q12.2 | q12.3 | 38 |
| chr18 | p11.32 | p11.23 | 49 |  |  |  |  | chr18 | q21.33 | q22.3 | 38 |
| chr18 | p11.22 | p11.21 | 49 |  |  |  |  | chr19 | p13.3 | p13.2 | 44 |
| chr18 | q11.2 | q11.2 | 49 |  |  |  |  | chr19 | p13.3 | p13.11 | 41 |
| chr18 | q11.2 | q12.3 | 51 |  |  |  |  | chr19 | q13.42 | q13.43 | 38 |
| chr18 | q12.1 | q12.2 | 49 |  |  |  |  | chr19 | q13.43 | q13.43 | 41 |
| chr18 | q21.1 | q23 | 49 |  |  |  |  | chr20 | p13 | p13 | 38 |
| chr18 | q21.1 | q21.2 | 54 |  |  |  |  | chr20 | p11.1 | q11.1 | 44 |
| chr18 | q21.2 | q23 | 51 |  |  |  |  | chr20 | q11.21 | q11.22 | 50 |
| chr18 | q21.31 | q23 | 54 |  |  |  |  | chr20 | q11.1 | q11.23 | 47 |
| chr18 | q22.1 | q22.3 | 57 |  |  |  |  | chr20 | q11.22 | q11.23 | 44 |
| chr18 | q22.2 | q22.3 | 60 |  |  |  |  | chr20 | q11.23 | q11.23 | 38 |
| chr18 | q22.3 | q23 | 57 |  |  |  |  | chr20 | q12 | q12 | 38 |
| chr20 | q11.1 | q11.23 | 51 |  |  |  |  | chr20 | q12 | q13.13 | 41 |
| chr20 | q12 | q13.2 | 49 |  |  |  |  | chr20 | q13.13 | q13.2 | 44 |
|  |  |  |  |  |  |  |  | chr20 | q13.2 | q13.33 | 38 |
|  |  |  |  |  |  |  |  | chr22 | q11.21 | q13.33 | 38 |

Green: gained region. Red: lost region.
